# Neutrophil-mediated IL-6 receptor trans-signaling and the risk of chronic obstructive pulmonary disease and asthma

**DOI:** 10.1093/hmg/ddx053

**Published:** 2017-02-17

**Authors:** Neda Farahi, Ellie Paige, Jozef Balla, Emily Prudence, Ricardo C. Ferreira, Mark Southwood, Sarah L. Appleby, Per Bakke, Amund Gulsvik, Augusto A. Litonjua, David Sparrow, Edwin K. Silverman, Michael H. Cho, John Danesh, Dirk S. Paul, Daniel F. Freitag, Edwin R. Chilvers

**Affiliations:** 1 Division of Respiratory Medicine, Department of Medicine, University of Cambridge School of Clinical Medicine, Cambridge CB2 0QQ, UK; 2 Department of Public Health and Primary Care, Strangeways Research Laboratory, University of Cambridge CB1 8RN, Cambridge, UK; 3 JDRF/Wellcome Trust Diabetes and Inflammation Laboratory, Nuffield Department of Medicine, Wellcome Trust Centre for Human Genetics, University of Oxford, Oxford OX3 7BN, UK; 4 Department of Clinical Science, University of Bergen, Bergen 5021, Norway; 5 Brigham and Women’s Hospital and Harvard Medical School, Boston 02115, MA, USA; 6 VA Boston Healthcare System and School of Medicine, Boston University, Boston 02132, MA, USA; 7 British Heart Foundation Centre of Excellence, University of Cambridge School of Clinical Medicine, Cambridge CB2 0QQ, UK; 8 NIHR Blood and Transplant Research Unit in Donor Health and Genomics, Cambridge, UK; 9 Wellcome Trust Sanger Institute, Hinxton CB10 1SA, UK

## Abstract

The Asp358Ala variant in the *interleukin-6 receptor* (*IL-6R*) gene has been implicated in asthma, autoimmune and cardiovascular disorders, but its role in other respiratory conditions such as chronic obstructive pulmonary disease (COPD) has not been investigated. The aims of this study were to evaluate whether there is an association between Asp358Ala and COPD or asthma risk, and to explore the role of the Asp358Ala variant in sIL-6R shedding from neutrophils and its pro-inflammatory effects in the lung. We undertook logistic regression using data from the UK Biobank and the ECLIPSE COPD cohort. Results were meta-analyzed with summary data from a further three COPD cohorts (7,519 total cases and 35,653 total controls), showing no association between Asp358Ala and COPD (OR = 1.02 [95% CI: 0.96, 1.07]). Data from the UK Biobank showed a positive association between the Asp358Ala variant and atopic asthma (OR = 1.07 [1.01, 1.13]). In a series of *in vitro* studies using blood samples from 37 participants, we found that shedding of sIL-6R from neutrophils was greater in carriers of the Asp358Ala minor allele than in non-carriers. Human pulmonary artery endothelial cells cultured with serum from homozygous carriers showed an increase in MCP-1 release in carriers of the minor allele, with the difference eliminated upon addition of tocilizumab. In conclusion, there is evidence that neutrophils may be an important source of sIL-6R in the lungs, and the Asp358Ala variant may have pro-inflammatory effects in lung cells. However, we were unable to identify evidence for an association between Asp358Ala and COPD.

## Introduction

Interleukin-6 (IL-6), a pro-inflammatory cytokine, has been implicated in a range of complex chronic diseases. Evidence has been building that one functional variant Asp358Ala (rs2228145) in the *IL-6 receptor* (*IL-6R*) gene regulates IL-6 signaling (1). Previous studies have shown that this variant is associated with a decreased risk of coronary heart disease (CHD) (2–4), abdominal aortic aneurysm (5), atrial fibrillation (6), rheumatoid arthritis (7) and type 1 diabetes (1), but an increased risk of atopic dermatitis (8) and asthma (9).

One hypothesis to explain the varying directional effects of the IL-6R variant on disease risk is based on the opposing effects of the variant on IL-6 classic signaling and trans-signaling. IL-6 signaling occurs via two distinct pathways, designated as ‘classical’ signaling and ‘trans-signaling’. In the classical signaling binding of IL-6 to the membrane bound IL-6 receptor (mIL-6R) causes recruitment of two molecules of the signal-transducing receptor glycoprotein 130 (gp130). Homodimerisation of the gp130 receptors leads to phosphorylation of transcription factors STAT1 or STAT3 by the Janus kinase family (JAK1, JAK2, TYK2), as well as activation of the mitogen-activated protein kinase (MAPK) cascade and the phosphoinositide 3-kinase (PI3K) cascade (10). These downstream pathways can also be activated by IL-6 trans-signaling via the generation of a soluble form of the IL-6 receptor (sIL-6R), which forms a complex with IL-6 and gp130. While sIL-6R can be released following alternative splicing of the IL-6R mRNA, the majority of circulating sIL-6R is generated by ADAM10- and ADAM17-mediated proteolysis of the mIL-6R (11). The substitution of alanine for aspartate in position 358 of the *IL-6R* gene results in more efficient proteolytic cleavage of mIL-6R (12). This results in decreased levels of mIL-6R, which is involved in classic signaling, and increased levels of soluble sIL-6R, which is involved in trans-signaling (1). IL-6R classic signaling occurs only in leukocytes and hepatocytes which express the mIL-6R (13), and there is evidence that the down-regulation of classic signaling in people with one or two copies of the minor allele (C) leads to the observed decreased risk of diseases such as CHD (1). In IL-6 trans-signaling, sIL-6R bound to IL-6 stimulates gp130 (13), which is widely expressed, including in lung cells. Since sIL-6R can trigger IL-6 signaling in cells normally unresponsive to IL-6, trans-signaling has been implicated in the pathogenesis of a number of inflammatory diseases.

Higher levels of sIL-6R have been found in sputum and bronchoalveolar lavage fluid (BALF) of people with asthma or chronic obstructive pulmonary disease (COPD) compared to healthy controls (14,15). Additionally, previous studies have shown associations between polymorphisms in the IL-6 gene and increased risk of COPD—although not at genome-wide levels of statistical significance (16). Combined with the replicated finding that rs2228145 is associated with asthma and asthma severity (9,17), this led us to hypothesize that the Asp358Ala functional variant may be associated with other respiratory conditions such as COPD. Current evidence suggests that neutrophils are involved in COPD and also occur in greater quantities in the BALF of people with severe asthma or smokers with asthma (18). Thus, we hypothesized that neutrophils are a possible source of sIL-6R in the lungs and may contribute to pathogenic IL-6R trans-signaling in chronic respiratory diseases.

In the present study, we first investigated the relationship between the IL-6R variant and the risk of COPD, using genetic data from the UK Biobank, a large-scale UK-wide prospective cohort study of middle-aged volunteers, along with several other well-phenotyped COPD cohorts with genetic information on the functional IL-6R variant and its proxies. Second, we sought to replicate the association between the IL-6R functional variant and an increased risk of asthma and allergy-related diseases using data collected from participants in the UK Biobank. Third, we conducted *in vitro* experiments to examine whether the IL-6R variant is associated with increased shedding of sIL-6R from neutrophils and whether IL-6R trans-signaling is pro-inflammatory in the lung micro-environment.

## Results

**Figure 1:**
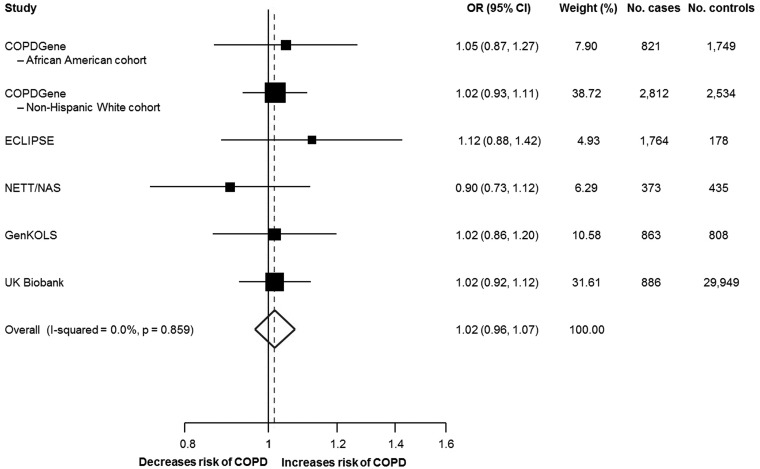
Forest plot showing the association between rs2228145 and the risk of COPD. CI =, confidence interval; COPD =, chronic obstructive pulmonary disease; OR =, odds ratio. Results are for current or former smokers and adjusted for age, ancestry principal components, and smoking amount. Fixed effects meta-analysis using inverse-variance weighting. Sizes of the boxes are proportional to the number of cases.

Since previous studies have shown higher levels of serum IL-6 in people with COPD compared to those without COPD (19–22), we first examined the association between rs2228145 and baseline serum IL-6 level in the ECLIPSE study, finding an increase in IL-6 level per copy of the minor allele after adjustment for age, COPD status, smoking status, smoking amount and ancestry (β = 0.15 [95% CI: 0.08, 0.23]). We found no evidence of an association between rs2228145 and COPD when the results from the UK Biobank (Supplementary Material, Table S1) and the COPD case-control cohorts were meta-analyzed (OR = 1.02 [0.96, 1.07]; Fig. 1). There was no evidence of heterogeneity between the studies contributing to the meta-analysis (I^2 ^=^ ^0.0%, *P* = 0.859). Results were consistent for the association between rs4129267 (proxy variant) and COPD (OR = 1.02 [0.95, 1.08]; Supplementary Material, Fig. S1), and also when meta-analyzed using random-effects meta-analysis (data not shown).

**Figure 2:**
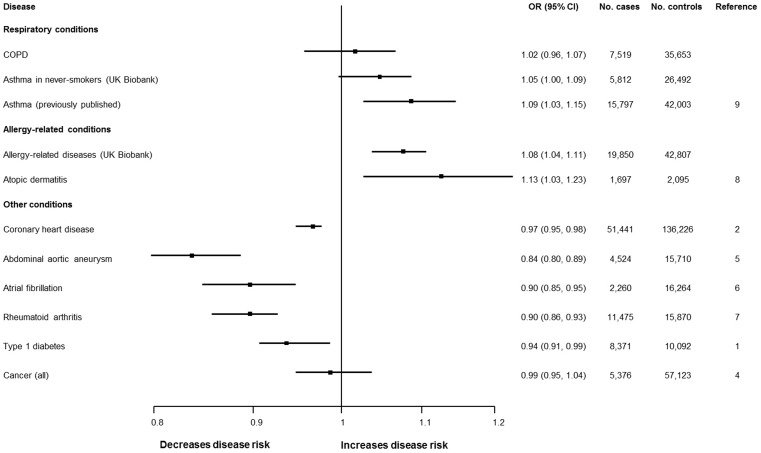
Forest plot showing associations of rs2228145 (or proxy) with a range of human di**seases**. CI =, confidence interval; COPD =, chronic obstructive pulmonary disease; OR =, odds ratio. OR plotted on log-scale.

Using UK Biobank data, we found no evidence of an overall association between rs2228145 and risk of asthma (OR = 1.02 [95% CI: 0.99, 1.05]; Table 1) but when comparing asthma phenotypes, we observed an increased risk of atopic asthma compared to non-atopic asthma per copy of the minor allele of rs2228145 (OR = 1.07 [1.01, 1.13]; Table 1). The rs2228145 variant was also associated with an increased risk of allergy-related disease phenotypes (OR = 1.08 [1.04, 1.11]; Table 2). These results persisted in analyses that used increasingly restrictive sets of exclusion criteria based on history of inflammatory-related illness (CHD, stroke, diabetes or cancer) and other respiratory illnesses (COPD, asthma (where relevant), pneumonia or bronchiectasis). The results of this new human genetic data on COPD and asthma risk in the UK Biobank population are shown in context of other known disease risks associated with rs2228145 in Figure 2.

**Table 1:** Adjusted odds ratios (95% CI) for the risk of asthma and atopic asthma per copy of the minor allele of rs2228145

	Overall asthma			Atopic vs. non-atopic asthma*		
	N cases/N controls	OR (95% CI)	*P*-value	N cases/N controls	OR (95% CI)	*P*-value
**Model 1**	16,939/83,718	1.02 (1.00, 1.04)	0.096	5,318/8,551	1.05 (1.00, 1.11)	0.047
**Model 2**	13,591/65,183	1.02 (1.00, 1.05)	0.075	4,550/6,607	1.07 (1.01, 1.13)	0.031
**Model 3**	13,157/63,859	1.02 (0.99, 1.05)	0.126	4,405/6,396	1.07 (1.01, 1.13)	0.032

CI =, confidence interval; CHD =, coronary heart disease; OR =, odds ratio. *Atopic asthma defined as age of asthma onset before 30 years and having either a history of allergic conditions (hay fever, allergic rhinitis and eczema) or having never smoked. Notes: (1) UK Biobank data only. (2) Results adjusted for sex, age, smoking status and ancestry principal components. (3) Model 1 includes the full dataset with no restrictions based on history of disease; Model 2 excludes people with inflammatory-related illness (history of CHD, stroke, diabetes or cancer); and Model 3 excludes people with inflammatory-related illness and those with other respiratory illnesses (chronic obstructive pulmonary disease, pneumonia or bronchiectasis).

**Table 2:** Adjusted odds ratios (95% CI) for the risk of allergy-related conditions per copy of the minor allele of the rs2228145

	N cases/N controls	OR (95% CI)	*P*-value
**Model 1**	24,112/57,643	1.06 (1.03, 1.08)	9.00 × 10^−7^
**Model 2**	20,223/43,796	1.07 (1.04, 1.09)	2.58 × 10^−7^
**Model 3**	19,850/42,807	1.08 (1.04, 1.11)	4.00 × 10^−6^

CI =, confidence interval; CHD =, coronary heart disease; OR =, odds ratio. Notes: (1) Allergy-related conditions include hay fever, allergic rhinitis and eczema. (2) UK Biobank data only. (3) Results adjusted for sex, age, BMI, smoking status, smoking amount and ancestry principal components. (4) Model 1 includes the full dataset with no restrictions based on history of disease; Model 2 excludes people with inflammatory-related illness (history of CHD, stroke, diabetes or cancer); and Model 3 excludes people with inflammatory-related illness (history of CHD, stroke, diabetes or cancer) and those with other respiratory illnesses (chronic obstructive pulmonary disease, asthma, pneumonia or bronchiectasis).

We further assessed whether the rs2228145 variant was associated with decreased airway function or with an increased risk of airway obstruction using spirometry measures from the UK Biobank. We found no evidence of an association between rs2228145 and airway function or obstruction, including when the results were stratified by asthma and COPD status and examined separately by smoking status (Supplementary Material, Tables S2 and S3).

**Figure 3:**
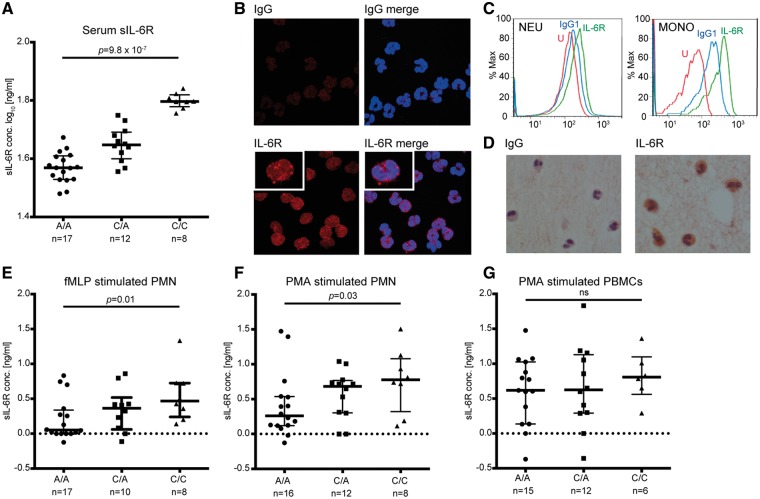
IL-6R alpha expression in human neutrophils and association of the rs2228145 genotype with secretion of sIL-6R by leukocytes. (**A**) Serum sIL-6R concentration from the rs2228145 genotype groups was measured by ELISA. (**B**) Immunofluorescence of IL-6R alpha AF647and IgG1 AF647 isotype matched control in neutrophils. Merged images show DAPI and IL-6R AF647 or IgG1 AF647. Magnification x63 with x5 zoom. (**C**) Expression of IL-6R alpha in neutrophils (NEU) and monocytes (MONO) analyzed by flow cytometry. Red (unstained), blue (IgG1) and green (IL-6R AF647). Monocytes were gated from the peripheral blood mononuclear cell population based on their forward scatter and side scatter properties. (**D**) Immunocytochemistry of IL-6R alpha and IgG1 isotype matched control in neutrophils. Magnification ×100. Data are representative of three independent experiments. (**E**) sIL-6R release from the rs2228145 genotyped polymorphonuclear neutrophils (PMN) stimulated for 30 min with fMLP or PMA (**F,G**) sIL-6R release from the rs2228145 genotyped peripheral blood mononuclear cells (PBMCs) stimulated for 30 min with PMA. sIL-6R concentrations refer to the difference between stimulated and control levels for B–D. Data represent the median with interquartile range. *P<0.05 using non-parametric test for trend across groups.

To elucidate the association of the Asp358Ala non-synonymous variant with asthma and allergy-related phenotypes, we next examined the molecular mechanism through a series of *in vitro* experiments. As previously shown, the Asp358Ala variant was associated with increased concentrations of sIL-6R in the serum (Fig. 3A). We then investigated the expression of mIL-6R in human peripheral blood-derived neutrophils. We demonstrated the expression of mIL-6R in freshly isolated neutrophils using confocal microscopy (Fig. 3B), flow cytometry (Fig. 3C) and immunocytochemistry (Fig. 3D). Expression of IL-6R in peripheral blood mononuclear cells (PBMCs) was used as a positive control for flow cytometry analysis. These results suggest that neutrophils can provide a source of sIL-6R in the plasma.

Given that previous work (23) has described shedding of IL-6R from stimulated neutrophils, we sought to compare sIL-6R release between rs2228145 C/C homozygous, A/C heterozygous and A/A homozygous individuals. Upon stimulation with either the physiological agonist *N*-formyl-methionyl-leucyl-phenylalanine (fMLP) or phorbol 12-myristate 13-acetate (PMA), sIL-6R release from neutrophils was significantly greater in C/C homozygous donors compared to A/A homozygous donors (Figs 3E and F). This effect was not observed in PBMCs (Fig. 3G). Taken together, these results are indicative of a genotype-dependent association of sIL-6R release from neutrophils.

**Figure 4:**
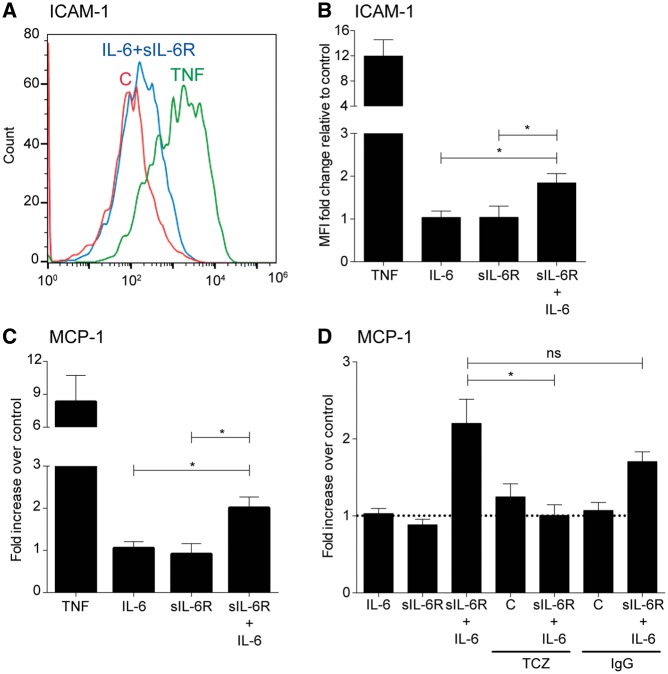
Effect of sIL-6R trans-signaling on ICAM-1 expression and MCP-1 release from HPAECs. Confluent human pulmonary endothelial cells (HPAECs) were stimulated for 24 h with TNF (5 ng/ml) or sIL-6R (50 ng/ml) ± IL-6 (50 ng/ml) prior to flow cytometry as described in Materials and Methods. (**A**) Representative flow cytometry of ICAM-1 expression in HPAECs after 24 h showing control (red), TNF (green) and sIL-6R + IL-6 (blue) treatments. (**B**) Median fluorescence intensity of ICAM-1 expression. Data are representative of six independent experiments (mean ± SEM). *P<0.05 using one-way ANOVA across groups. (**C**) Confluent HPAECs were stimulated for 24 h with TNF (5 ng/ml) or sIL-6R (50 ng/ml) ± IL-6 (50 ng/ml) prior to measurement of secreted MCP-1 by ELISA. Data are representative of five independent experiments (mean ± SEM). *P<0.05 using Dunn’s multiple comparison test. (**D**) Confluent HPAECs were stimulated for 24 h with sIL-6R (50 ng/ml) ± IL-6 (50 ng/ml), tocilizumab (TCZ) alone (20 μg/ml), IgG alone (20 μg/ml) or IL-6 + sIL-6R (pre-incubated with 20 μg/ml TCZ or 20 μg/ml IgG) and MCP-1 measured by ELISA. Data are representative of eight independent experiments (mean ± SEM). * *P*<0.05 using Dunn’s multiple comparison test.

IL-6 trans-signaling has been previously identified as pro-inflammatory in smooth muscle cells, mesothelial cells and endothelial cells (24–26). To assess the effect of sIL-6R trans-signaling in the lung endothelium, we measured the expression of various cell adhesion markers and monocyte chemoattractant protein-1 (MCP-1) release by human pulmonary artery endothelial cells (HPAECs) following treatment with recombinant IL-6 and sIL-6R alone or in combination. TNF-α treatment was used as a positive control. Stimulation with either IL-6 or sIL-6R alone did not alter intercellular adhesion molecule 1 (ICAM-1) levels detected by flow cytometry after 24 h (Fig. 4A). A stimulation period of 24 h was chosen as the MCP-1 release by HPAECs (using IL-6 and sIL-6R) was demonstrated to be maximal at this time-point (data not shown). However, IL-6 in combination with sIL-6R significantly increased the expression of ICAM-1 and the release of MCP-1 by ∼ 2-fold in HPAECs (Figs 4B and C). The MCP-1 release was suppressed in the presence of the anti-human IL-6R monoclonal antibody tocilizumab (Fig. 4D). There was no change in the expression of vascular cell adhesion protein 1 (VCAM-1) and E-selectin (Supplementary Material, Fig. S2). Human bronchial epithelial cells did not enhance MCP-1 (data not shown) or IL-8 release (Supplementary Material, Fig. S3) following combined IL-6 and sIL-6R stimulation.

**Figure 5:**
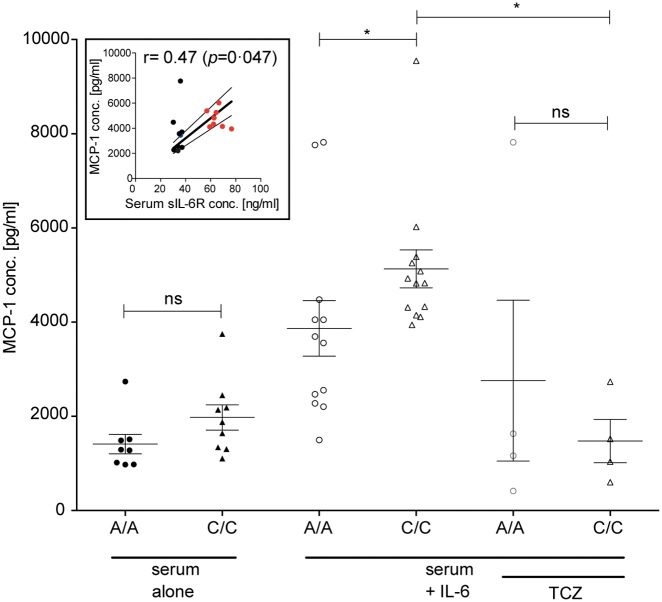
Serum from Asp358Ala carriers increases MCP-1 release from HPAECs. Confluent HPAECs were cultured for 24 h in the presence of 65% A/A or C/C serum ± IL-6 (50 ng/ml) prior to measurement of secreted MCP-1 by ELISA. Data are presented as individual values and means ± SEM. *P<0.05 using Mann-Whitney test. The inset shows the association of serum sIL-6R concentration with IL-6 mediated MCP-1 release from HPAECs. Data are presented as individual values for 8 C/C (red circles), 1 C/A (blue circle) and 9 C/C (black circles) samples. Correlation calculated using the Spearman test with the 95% confidence band of the best-fit line.

Having shown the pro-inflammatory effect of IL-6 trans-signaling in the lung endothelium, we next sought to test our hypothesis that increased sIL-6R shedding by C/C individuals is pro-inflammatory. We cultured HPAECs with serum from 8 A/A and 9 C/C participants and measured MCP-1 release after 24 h. As shown in Figure 5, incubation with serum from C/C carriers resulted in no increase in the MCP-1 release compared to A/A carriers (A/A serum; 1334 ± 195.6 pg/ml and C/C serum; 1977 ± 268.1 pg/ml, *P *=* *0.06). The addition of A/A serum alone resulted in MCP-1 concentrations comparable to that of untreated cells at 24 h (1030 ± 319.7 pg/ml), suggesting that addition of serum did not adversely affect the HPAECs. The addition of IL-6 to the serum resulted in the increased MCP-1 release in both groups compared to serum alone, but significantly more MCP-1 release from C/C carriers (A/A serum; 3604 ± 579.7 pg/ml and C/C serum; 4761 ± 232.4 pg/ml, *P *=* *0.009). This response was also replicated using lower IL-6 concentrations (10 ng/ml) (Supplementary Material, Fig. S4A). There were no statistically significant differences in IL-6 or MCP-1 concentrations between the C/C and A/A serum prior to their addition to HPAECs (Supplementary Material, Fig. S4B and C).Furthermore, incubation with tocilizumab reversed the IL-6 mediated MCP-1 release in both groups to baseline levels and, importantly, abolished the difference between the A/A and C/C carriers (Fig. 5).

## Discussion

Although there is considerable evidence that levels of sIL-6R are increased in people with respiratory diseases such as asthma and COPD, the genetic association between the Asp358Ala functional variant in the IL-6R gene and COPD has not been specifically assessed, and little is understood about the mechanisms driving sIL-6 trans-signaling in the lung. In this study, we provide further evidence for the association of the Asp358Ala non-synonymous variant with atopic asthma and a broad group of allergy-related conditions, but not with COPD risk. Most importantly, we show, for the first time, an association between the Asp358Ala variant and increased shedding of sIL-6R from human neutrophils and provide evidence for a pro-inflammatory effect of sIL-6 trans-signaling in human pulmonary artery endothelial cells. We observed an increased release of MCP-1 (a chemokine involved in inflammatory responses) from lung endothelial cells after addition of serum from homozygous carriers of the minor allele but not after addition of serum from homozygous carriers of the major allele. This effect was blocked with the addition of the anti-IL-6R monoclonal antibody tocilizumab, suggesting that the observed genotype-dependent release of MCP-1 occurred via IL-6 signaling pathways and not through an alternative mechanism.

In contrast to our hypothesis, we found no association between the Asp358Ala variant and the risk of COPD. This was surprising given reports of increased levels of sIL-6R in people with COPD (15), and previous reports of associations of polymorphisms in the IL-6 gene with this condition (16,27,28). Recent experimental evidence in mouse models supports the involvement of IL-6 trans-signaling in the development of emphysema via activation of the mammalian target of rapamycin complex 1 (mTORC1) pathway and that this is independent of the inflammatory response (29). Based on this and previous work (30), the authors hypothesized that IL-6R trans-signaling may contribute to lung inflammation and the development of emphysema via two discrete pathways. Firstly, they propose that sIL-6R acts via STAT3 activation to initiate lung inflammation (30) and, secondly, through a pathway whereby sIL-6R activates mTORC1 and contributes to the development of emphysema (29). It is known that the Asp358Ala variant increases sIL-6 signaling, which activates STAT3, a necessary component for inflammatory processes, via the JAK-STAT and Ras-Raf-MAPK signaling pathways (31), however we are not aware of evidence that the Asp358Ala variant directly activates the mTORC1 pathway. Furthermore, a previous genome-wide association study (32) found no evidence of an association between the rs2228145 variant and emphysema defined using lung density images using the traditional definition of −950 Hounsfield units with *P* value = 0.192, and borderline nominal significance for the 15th percentile of the density histogram (*P* value = 0.042).

There are two possible explanations for the results of our study when considered in the context of this previous experimental work. If the STAT3 and mTORC1 pathways are independent, as has been suggested (30), then activation of the mTORC1 pathway downstream of sIL-6R may require additional signals over and above increased levels of sIL-6R. This could explain why we found no evidence of an association between the Asp358Ala variant and the risk of COPD, and why the previous study (32) found no association between this same variant and emphysema. That is, the variant might act as an instrumental variable for exposure to increased levels of sIL-6R only. Alternatively, our results could result from a lack of power to detect an effect size as small as that observed. Our study was only powered to find an odds ratio of greater than 1.075, so if enhanced IL-6 trans-signaling does contribute to the pathogenesis of COPD, it is likely that this relationship is weaker than that observed for asthma in previous studies and may therefore be of limited clinical relevance.

In contrast to a previous genome-wide association study that reported a 9% increased risk of asthma for carriers of the minor allele of the rs4129267 variant (proxy for rs2228145) (9), we found no overall association between the rs2228145 and asthma (OR = 1.02 [0.99, 1.05]). Our study was powered to see an effect size of 1.055 or greater, suggesting that the effect of this variant on asthma was weaker in our study compared to this previous study. However, it was also noted in the study by Ferreira and colleagues (9) that this association appeared to be restricted to atopic asthma, and our finding of an increased risk of atopic asthma vs. non-atopic asthma (albeit using a proxy measure) among carriers of the rs2228145 minor allele adds some weight to these previous unconfirmed findings. We did not have information on specific asthma phenotypes and instead we used a carefully defined proxy for atopic asthma (based on age of onset, history of allergies and smoking status (33)). Further, our finding that the Asp358Ala variant is associated with an increased risk of allergy-related diseases is consistent with a previous study showing an increased risk atopic dermatitis (8). In contrast to a previous study (17), we found no association between the Asp358Ala variant and decreased lung function in asthmatic never-smokers, potentially due to differences in asthma phenotyping which was more rigorously characterized using spirometry measures and bronchodilator reversibility in the previous study.

Previous studies have shown sIL-6R levels in serum are increased in people with asthma, particularly among those with severe asthma phenotypes, and these sIL-6R levels are associated with decreased lung function (17). Neutrophils have been identified as a potential source of sIL-6R in asthma (13). Consistent with this hypothesis and with a previous study, which showed that showed neutrophils can be stimulated to shed sIL-6R in response to inflammatory cues (23), we have directly shown that neutrophils shed sIL-6R and that this shedding is increased in carriers of the Asp358Ala minor allele. Although endothelial cells do not express the mIL-6R required for classic signaling, they do express gp130 and can utilize sIL-6R (trans-signaling) to secrete monocyte MCP-1, IL-8 and induce ICAM-1 expression (26,34). Our finding that IL-6/sIL-6R trans-signaling increases MCP-1 release and ICAM-1 expression in lung endothelial cells is consistent with findings from previous studies that used human umbilical vein endothelial cells (34). In addition to aiding monocyte recruitment, MCP-1 is known to induce mast cell activation and LTC_4_ release in the airway (35), and clinical studies have demonstrated that MCP-1 is up-regulated during the endobronchial allergen challenge (36). Our study thus extends previous work by showing that MCP-1 release from lung endothelial cells is increased in carriers of the Asp358Ala minor allele and can be blocked using the IL-6 inhibitor tocilizumab, providing evidence of a pro-inflammatory effect of IL-6 trans-signaling in the lung micro-environment. The only previous study to examine the functional effect of the minor variant on monocytes reported a genotype-dependent effect on unstimulated PBMCs (13). In contrast, we found no genotype-dependent effect on sIL-6R release from PBMCs; the reason for this discrepancy is unclear but may reflect the larger sample size in this study or differing PBMC isolation methods.

This is the first study, to our knowledge, to specifically assess the relationship of the Asp358Ala variant with the risk of COPD, and the first to describe the associations of this variant with enhanced sIL-6R shedding from neutrophils and enhanced MCP-1 release from human lung endothelial cells. The COPD cohorts were well-phenotyped and the genetic analysis results were robust to sensitivity analyses, including restricting to studies with data on the proxy variant rs4129267 (i.e. excluding UK Biobank, the least well-phenotyped study included). As well as these strengths, our study has some limitations. Although a large number of COPD cases (*n* = 7,519) and asthma cases (*n =* 13,157) were included in the analysis, the study only had 80% power to detect an odds ratio of 1.075 or more for COPD and 1.055 or more for asthma. In the genetic analysis, only summary-level data could be obtained for four out of six of the studies; however, we were able to closely match the exclusion criteria and variables adjusted for in the statistical analysis of the two studies with individual participant data (ECLIPSE and UK Biobank) to those used in the other four studies. Although there was no evidence of heterogeneity between the studies contributing to the Asp358Ala variant-COPD analysis, it is notable that the direction of the relationship was the same for all cohorts except for NETT/NAS. The NETT cohort differs from the other COPD cohorts in that only patients with both more severe COPD and emphysema were recruited (37); thus the included participants are likely to represent a more restricted sub-phenotype of COPD compared to the other cohorts. Disease outcomes in the UK Biobank were based on self-reported data which can be susceptible to misclassification. However, for the main outcome of COPD, we were able to minimize misclassification by only including COPD cases where the self-report was either verified in an interview with a trained nurse or where there was a hospital admission diagnosis of COPD. Further, we used objective spirometry measures to exclude those with self-reported COPD who had normal lung function (defined as a ratio of forced expiratory volume in one second (FEV1)/forced vital capacity (FVC) ≥ 0.7 and a percent predicted FEV1 ≥ 80). Analyses of airway function, airway obstruction, asthma and allergy-related conditions were restricted to UK Biobank data as information on these outcomes was not available in the COPD cohorts.

There is strong evidence that inflammation is involved in the etiology of a number of chronic diseases, yet despite this and existing evidence for the role of IL-6 in COPD (15), our study shows no strong evidence for an association between inflammation via IL-6 trans-signaling and the risk of COPD. This is potentially concordant with recent experimental evidence that suggests the role of IL-6 trans-signaling in the development of emphysema occurs independently of inflammatory processes (29), although replication of the results is needed. Furthermore, while there is strong evidence that IL-6 serum levels are increased in COPD patients compared to healthy controls, there is no evidence of an exposure-response relationship with disease severity (15) as would be expected in a causal relationship and as is observed for asthma (17).

More generally, our results, taken together with those from previous studies, suggest that IL-6 signaling plays a role in allergic inflammation, potentially contributing to the development of atopic asthma phenotypes and other allergy-related diseases. Mechanistically, this finding is supported by a number of studies using mouse models and patient samples. In particular, Doganci and colleagues (38) found that local blockade of the sIL-6R in an ovalbumin mouse model of the late-phase asthma response reduced eosinophil numbers in BALF and decreased levels of the Th2 cytokines IL-4, IL-5, and IL-13. In a further mouse model, inhalation of cockroach allergen resulted in an increase in sIL-6R and triggered a type 2/type 17 cytokine proﬁle with mixed eosinophilic-neutrophilic inﬂammation in BALF, a response that was attenuated using an anti-IL-6R molecule that selectively blocks IL-6 trans-signaling (39). In humans with allergic asthma, sputum sIL-6R levels have been shown to be correlated with the number of IL-5 producing CD4+ T cells following allergen challenge and IL-6 levels in sputum have been shown to be associated with a mixed eosinophilic-neutrophilic bronchitis during exacerbations of asthma (38,40). Furthermore, Ullah and colleagues (39) have shown that asthmatics with high (greater than median) levels of IL-6 and IL-6R in the airways were more likely to have neutrophilic rather than eosinophilic asthma profiles.

Our findings of enhanced shedding of sIL-6R from neutrophils of Asp358Ala homozygotes and an increase in the MCP-1 release from lung endothelial cells using serum from these individuals provides evidence of a mechanism through which IL-6 trans-signaling may contribute to disease severity in severe, neutrophil-driven, asthma phenotypes. The IL-6 signaling pathway is an important therapeutic target for rheumatoid arthritis and Castleman’s disease (41,42), and is being investigated in clinical trials for other diseases including systemic lupus erythematosus (43). Our results support research into the potential for targeting this pathway to treat allergic inflammatory conditions, such as severe asthma, while future research with larger numbers of COPD cases is needed to understand more fully the role of IL-6 trans-signaling and its associated pathways in the development of COPD.

## Materials and Methods

### Genetic epidemiological study

*Participants* *.* Five cohorts contributed data to this study. Data from participants in the ECLIPSE study were used to estimate the association between the rs2228145 variant and baseline serum IL-6 level, and the risk of COPD. Details on this study have been previously published (44). Briefly, ECLIPSE is a three-year longitudinal study with data on 2164 people with well-phenotyped COPD and 582 controls (337 smokers and 245 non-smoker controls). Data on 1,942 participants (1764 people with COPD and 178 smoker controls) with imputed genetic data on the rs2228145 and genotyped data on a proxy variant (rs4129267) were used in the present study. A summary of baseline characteristics of the ECLIPSE participants by rs2228145 genotype is given in Supplementary Material, Table S4. Data from participants in the UK Biobank were used to estimate the association between the rs2228145 and asthma, allergy-related diseases, airway function, airway obstruction, and the risk of COPD. The UK Biobank is an open-access resource with survey, biomedical and genetic data, and linked hospital episode statistics data, on approximately 500,000 people aged 40 to 69 years at baseline living in the UK. Genotyped data on rs2228145 from 134,388 participants were available for this study. Details on the study measures and ascertainment of outcomes in the UK Biobank are provided in S1 Appendix, and details on genotyping and genetic QC are available online (45). This research has been conducted using the UK Biobank Resource under Application Number 20480. Participants provided written informed consent to be involved in the cohort. A summary of baseline characteristics of the UK Biobank participants by rs2228145 genotype is given in Supplementary Material, Table S5. Summary statistics for the association between rs2228145 (and proxy rs4129267) and the risk of COPD were also available for the COPDGene, the National Emphysema Treatment Trial (NETT)/Normative Aging Study (NAS), and GenKOLS studies (37,46–49).

#### Statistical analyses

Linear regression was used to estimate the association between the rs2228145 variant and log-transformed baseline level of serum IL-6 in the ECLIPSE study with adjustment for age, COPD status, smoking status, smoking amount and ancestry principal components. Logistic regression was used to estimate the association between the SNPs (rs2228145 and rs4129267) and COPD separately in each study. The odds ratios (OR) show the change in risk of COPD per copy of the minor allele and are presented with 95% confidence intervals (95% CI). Analyses were restricted to former or current smokers and were adjusted for age, smoking amount and ancestry principal components. A series of exclusion criteria were applied to the UK Biobank data to reflect the exclusion criteria used in the other four studies. This involved excluding participants with inflammatory-related illness (history of CHD, stroke, diabetes, or cancer) or other respiratory conditions (history of asthma, pneumonia, or bronchiectasis). Effect estimates and standard errors were pooled using inverse-variance weighted fixed-effects meta-analysis. A sensitivity analysis was undertaken, pooling results using random-effects meta-analysis. Heterogeneity in ORs between studies was quantified using the I^2^ statistic (50).

Using data from the UK Biobank only, we also examined the association between rs2228145 and lung function (measured as the ratio of forced expiratory volume in one second (FEV1) to forced vital capacity (FVC)) using linear regression, and the associations between rs2228145 and binary outcomes of airflow obstruction (as per definition in (51)), asthma and atopic asthma, and allergy-related diseases (hay fever, allergic rhinitis and eczema) using logistic regressions. A proxy for atopic asthma was defined as having an asthma diagnosis before the age of 30 years (52) and having either a history of allergic conditions or being a never smoker. These factors have been previously shown to be predictors of atopic vs. non-atopic asthma (33). The effect sizes show the change in outcome per copy of the minor allele and are presented with 95% CIs. Analyses were adjusted for sex, age, smoking status, smoking amount and ancestry principal components.

Power calculations using the Quanto software (53) were undertaken to estimate the power available for the main analysis. All other analyses were done using Stata 14.2.

### *In vitro* study

*Participants* *.* Thirty seven healthy volunteers were recruited in the Department of Medicine, Addenbrookes’ Hospital. The age of blood donors ranged from 22 to 61 years-old and included 17 females and 20 males. All participants gave written informed consent and the study was approved by the Cambridgeshire 2 Research Ethics Committee (06/Q0108/281).

*Reagents* *.* All chemicals were from Sigma-Aldrich (Dorset, UK), unless otherwise stated. Recombinant sIL-6R and IL-6 were from PeproTech (London, UK) and tocilizumab (RoActemra, Roche, Welwyn Garden City, UK). Cell culture reagents were from Gibco (Paisley, UK).

*Genotyping* *.* Blood samples were genotyped by LGC Genomics at rs2228145 using competitive PCR-based KASP^TM^ technology.

#### Leukocyte isolation and stimulation

Neutrophils and peripheral blood mononuclear cells (PBMCs) were isolated from human venous blood using discontinuous plasma-Percoll gradients as described previously [8] before resuspension in IMDM. Cells (20 × 10^6^/ml) were stimulated for 30 min at 37 °C with fMLP (100 nM) or PMA (200 nM). Supernatants were collected, centrifuged for 5 min (1200g) and stored at −80 °C prior to ELISA measurement.

#### ELISAs

Human MCP-1 and CXCL8 (IL-8) were measured using matched antibodies from R&D Systems. Soluble IL-6R was measured using Europium-Streptavidin as previously described (1). Serum samples were collected in clot activating tubes (Vacutainer, BD, UK). IL-6 and MCP-1 in serum was measured using the V-plex human assay kit (Meso Scale Discovery, MD, Maryland, US) with a lower limit of detection of 0.06 pg/ml and 0.13 pg/ml respectively.

*Lung endothelial and epithelial cells* *.* Human pulmonary artery endothelial cells (HPAECs) were from Lonza (Slough, UK) and stimulated TNF-α (5 ng/ml) or sIL-6R ± IL-6 under growth-factor-free conditions for 24 h in EBM-2 media (Lonza, Switzerland). Serum stimulations were performed in EBM-2 media with 65% serum from A/A or C/C genotyped donors. Human bronchial epithelial cells (HBECs) were originally derived in the laboratory of Professor Jerry Shay, and with his permission were gifted to us by Professor Gisli Jenkins (Nottingham Respiratory Research Unit, Nottingham City Hospital). The HBECs were incubated under growth-factor free conditions for 4-24 h with TNF-α (5 ng/ml) or sIL-6R ± IL-6 prior to collection of supernatants for measurement. For the serum stimulation experiments 65% serum (v/v) was added to each well to obtain a final sIL-6R concentration as close to 50 ng/ml in the C/C donors.

#### Flow cytometry

Trypsinised endothelial cells were collected into a 96 well U-bottom plate and centrifuged at 400g for 5 min at room temperature. Cells were resuspended in blocking buffer (5% FBS in 0.5% BSA) for ≥10 min at 4 °C, and incubated in the dark with conjugated antibodies (E-selectin-Fluorescein (R&D Systems, UK); VCAM-1-PECY5 (Biolegend, UK); ICAM-1-APC (BD Biosciences, UK) or conjugated isotype controls (from R&D Systems, Biolegend and BD Biosciences respectively) for 30 min at 4 °C. Stained cells were centrifuged at 400g for 5 min at room temperature, and fixed in 1% paraformaldehyde (PFA) in PBS in the dark at room temperature for 10 min. Fixed cells were centrifuged at 400g for 5 min at room temperature, and resuspended in 400 μl staining buffer (1% FBS and 0.09% sodium azide in PBS). Detection of cell-bound conjugated antibodies was performed using a BD FACSCanto™ II (BD Biosciences, UK) and quantification was performed using FlowJo software (Tree Star, USA). Data were acquired from at least 10,000 gated events.

Expression of IL-6R in neutrophils and PBMCs was determined using IL-6R alpha AF647. Briefly, neutrophils or PBMCs (5 × 10^6^/ml) were fixed with CellFIX (BD, Biosciences, UK) for 1 min on ice before incubation with IL-6R alpha AF647 or isotype control (Biolegend, UK) for 30 min in the dark at 4 °C. Data were acquired from at least 10,000 gated events.

#### Immunofluorescence and immunocytochemistry

Neutrophils and PBMCs were fixed in 3.5% paraformaldehyde before three washes in PBS. Cells were cytocentrifuged onto polylysine slides, permeabilised with Triton-X-100 (0.5%) for 10 min and rinsed in PBS. Cells were blocked with 0.5% BSA for 30 min before incubation with antibody sIL-6R AF or isotype control and staining with DAPI based mountant. Immunofluorescence was visualized using a fluorescent microscope.

For immunocytochemistry pelleted neutrophils and PBMCs were placed into induced plasma/thrombin clots, formalin-fixed overnight and processed into paraffin wax block. 4µm sections were placed onto adhesive slides and high pH antigen retrieval performed using a Dako PT Module. Slides were washed in PBS-Tween, blocked with Hydrogen Peroxidase solution (Dako, UK) and stained with IL-6R alpha antibody (R&D Systems) or mouse IgG_1_ isotype (R&D Systems) control for 1 h at room temperature. Slides were washed as before, incubated with ENVision Flex HRP (Dako, UK) for 30 min and visualized with 3,3’-Diaminobenzedine (Dako, UK) to create a brown reaction product, counterstained with haematoxylin and examined by light microscopy.

#### Statistical analysis

Data are reported as mean ± standard error of the mean (SEM) from (n) independent experiments and analyzed using GraphPad Prism 6.0d software (GraphPad Prism, USA). A *P* value <0.05 was considered statistically significant.

## Supplementary Material

Supplementary Data
