# Supplementary material for: Neutrophil-mediated IL-6 receptor trans-signaling and the risk of chronic obstructive pulmonary disease and asthma
Source: Hum Mol Genet. 2017 Feb 17;26(8):1584–96. doi: 10.1093/hmg/ddx053 (PMC5393150; doi:10.1093/hmg/ddx053)
Supplement: Supplementary Data [file ddx053_Supp.pdf]

NEUTROPHIL-MEDIATED IL-6 RECEPTOR TRANS-SIGNALLING AND THE RISK OF CHRONIC OBSTRUCTIVE PULMONARY DISEASE AND ASTHMA

SUPPLEMENTARY MATERIAL

CONTENTS

S1 Appendix: Study measures and ascertainment of outcomes in the UK Biobank 2

S1 Table: Adjusted odds ratios (95% CI) for the risk of COPD per copy of the minor allele of rs2228145, stratified by smoking status 4

S2 Table: Change in airway function per copy of the minor allele of rs2228145, stratified by COPD and asthma status 5

S3 Table: Adjusted odds ratios (95% CI) for the risk of airway obstruction per copy of the minor allele of rs2228145, stratified by COPD and asthma status 5

S4 Table: Summary of baseline characteristics of ECLIPSE participants by genotype of rs2228145 6

S5 Table: Summary of baseline characteristics of UK Biobank participants by genotype of rs2228145....................6

S1 Figure: Forest plot showing the association between rs4129267 and the risk of COPD 7

S2 Figure: Effect of sIL-6R trans-signaling on E-selectin, VCAM-1 expression and IL-8 release..............................8

S3 Figure: Effect of sIL-6R trans-signaling on IL-8 release by HBECs 9

S4 Figure: Serum from Asp358Ala carriers increases MCP-1 release from HPAECs... 10

S1 Appendix: Study measures and ascertainment of outcomes in the UK Biobank

Quality control of UK Biobank genetic data

Quality control of the UK Biobank genetic data is published on the UK Biobank website (http://www.ukbiobank.ac.uk/wp-content/uploads/2014/04/UKBiobank_genotyping_QC_documentation-web.pdf). In total, 153,293 samples were genotyped across 33 batches. Samples with high missingness or high heterozygosity (accounting for ethnicity) were excluded based on visual inspection of ancestry-specific plots, as were samples from participants who had withdrawn. A further eight samples who had low heterozygosity that couldn't be explained by long runs of homozygosity were also excluded. For variants with multiple probesets, the probeset defined by Affymetrix as "best" was retained. Variants showing batch effects, within-batch plate effects, or within-batch deviations from Hardy-Weinberg equilibrium (HWE) in European ancestry samples defined by principal component analysis (PCA), all at p-value<1x10-12, were filtered from the batches in which they failed. Samples with more than 3% missingness, samples with missing phenotypic sex, and samples with sex mismatches or dubious sex estimation from the genotyped data were excluded. Analyses using UK Biobank data were restricted to non-related Caucasian Europeans with genotyped data on the rs2228145 variant.

Ascertainment of study outcomes

Asthma: This was ascertained using two methods: (1) self-report response to the survey question (given as a touch-screen medical questionnaire): "Has a doctor ever told you that you have had any of the conditions below?" where one of the included responses was "asthma"; (2) if asthma was coded during an interview with a trained nurse who asked participants whether they had any other conditions they had not listed on the survey and asked questions to verify the conditions reported in the medical questionnaire.

Allergy-related conditions: This was ascertained using two methods: (1) self-report response to the survey question (given as a touch-screen medical questionnaire): "Has a doctor ever told you that you have had any of the conditions below?" where one of the included responses was "hayfever, allergic rhinitis or eczema"; (2) if these conditions were coded during an interview with a trained nurse who asked participants whether they had any other conditions they had not listed on the survey and asked questions to verify the conditions reported in the medical questionnaire.

Chronic obstructive pulmonary disease (COPD): Ascertained using a combination of self-report, Nurse's interview, and measured spirometry. Similar to the approach used for asthma and allergy-related conditions, the self-report component was ascertained using two methods: (1) self-report response to the survey question (given as a touch-screen medical questionnaire): "Has a doctor ever told you that you have had any of the conditions below?" where one of the included responses was "emphysema/chronic bronchitis"; (2) if "chronic obstructive airways disease/copd", "emphysema/chronic bronchitis", "bronchitis", or "emphysema" were coded during an interview with a trained nurse who asked participants whether they had any other conditions they had not listed on the survey and verified those conditions reported in the medical questionnaire. Using linked hospital episode statistics (HES) data, participants were coded as having COPD if they had a diagnosis of COPD (ICD-10 codes: J40-J44) before or on the date of the baseline survey. Only participants who had COPD verified in the Nurse's interview, or a HES diagnosis of COPD were included as COPD cases in the current analysis. As there may be some misclassification using the self-report responses, participants who had self-report COPD but had normal lung function defined using the spirometry measurements were excluded from the analyses using COPD as the outcome. Normal lung function was defined as a ratio of forced expiratory volume in one second (fev1)/forced vital capacity (fvc)≥0.7 and percent predicted fev1≥0.8. Percent predicted fev1 was calculated using sex-specific equations from [1] that took into account participant height and age.

Airway function: Ascertained using spirometry measurements and defined as fev1/fvc. Airway function was modelled as a continuous variable.

Airway obstruction: Ascertained using spirometry measurements and defined as fev1<lower normal limits and fev1/fvc< lower normal limits. Lower normal limits were calculated using sex-specific equations from [2] and accounted for age and height. Airway obstruction was modelled as a binary variable.

References

1. Crapo RO, Morris AH, Gardner RM. Reference spirometric values using techniques and equipment that meet ATS recommendations. The American review of respiratory disease. 1981;123(6):659-64.
2. Quanjer PH, Tammeling GJ, Cotes JE, Pedersen OF, Peslin R, Yernault JC. Lung volumes and forced ventilatory flows. The European respiratory journal. 1993;6 Suppl 16:5-40.

S1 Table: Adjusted odds ratios (95% CI) for the risk of COPD per copy of the minor allele of rs2228145, stratified by smoking status

	Former or current smokers			Never smokers		
	**N cases/ N controls**	**OR (95% CI)**	**p-value**	**N cases/ N controls**	**OR (95% CI)**	**p-value**
**Model 1**	2,115/43,991	1.00 (0.94, 1.06)	0.921	596/55,373	1.09 (0.97, 1.22)	0.153
**Model 2**	1,422/34,202	0.98 (0.91, 1.06)	0.683	461/46,912	1.09 (0.96, 1.24)	0.184
**Model 3**	886/29,949	1.02 (0.92, 1.12)	0.753	199/40,529	1.05 (0.85, 1.28)	0.647

Notes: (1) UK Biobank data only. (2) Results adjusted for age, smoking status, smoking amount and ancestry principal components. (3) Model 1 includes the full dataset with no restrictions based on history of disease; Model 2 excludes people with inflammatory-related illness (history of CHD, stroke, diabetes or cancer); and Model 3 excludes people with inflammatory-related illness (history of CHD, stroke, diabetes or cancer) and those with other respiratory illnesses (asthma, pneumonia or bronchiectasis). COPD=chronic obstructive pulmonary disease

S2 Table: Change in airway function per copy of the minor allele of rs2228145, stratified by COPD and asthma status

	Never smokers			Former or current smokers		
	N	Change in airway function (95% CI)	p-value	N	Change in airway function (95% CI)	p-value
**All participants**	46,508	-0.000 (-0.001, 0.000)	0.321	39,422	-0.000 (-0.001, 0.001)	0.504
**Asthma**						
**No**	27,338	0.000 (-0.001, 0.001)	0.974	25,879	0.000 (-0.001, 0.001)	0.976
**Yes**	6,346	-0.001 (-0.004, 0.002)	0.480	4,987	-0.003 (-0.006, 0.001)	0.118
**COPD**						
**No**	45,729	-0.000 (-0.001, 0.000)	0.303	37,299	-0.000 (-0.001, 0.001)	0.985
**Yes**	396	0.002 (-0.011, 0.016)	0.746	1,592	-0.002 (-0.009, 0.005)	0.570

Notes: (1) UK Biobank data only. (2) Results adjusted for sex, age, smoking status, smoking amount and ancestry principal components. (3) Airway function defined as the ratio of fev1/fvc. COPD=chronic obstructive pulmonary disease

S3 Table: Adjusted odds ratios (95% CI) for the risk of airway obstruction per copy of the minor allele of rs2228145, stratified by COPD and asthma status

	Never smokers			Former or current smokers		
	N cases/ N controls	OR (95% CI)	p-value	N cases/ N controls	OR (95% CI)	p-value
**All participants**	2,060/38,309	1.01 (0.94, 1.07)	0.858	4,303/28,464	0.99 (0.95, 1.04)	0.817
**Asthma**						
**No**	779/22,881	0.95 (0.85, 1.05)	0.290	2,630/18,695	0.99 (0.93, 1.05)	0.687
**Yes**	1,086/3,864	1.04 (0.94, 1.16)	0.406	1,162/2,762	1.04 (0.94, 1.16)	0.432
**COPD**						
**No**	1,896/37,868	1.00 (0.93, 1.07)	0.937	3,318/27,787	0.97 (0.92, 1.03)	0.343
**Yes**	148/98	1.29 (0.87, 1.92)	0.208	972/194	0.97 (0.77, 1.22)	0.765

Notes: (1) UK Biobank data only. (2) Results adjusted for sex, age, BMI, smoking status, smoking amount and ancestry principal components. (3) Airway obstruction defined as fev1 and fev1/fvc less than the estimated normal lower limits for each. CI=confidence interval; COPD=chronic obstructive pulmonary disease; OR=odds ratio

S4 Table: Summary of baseline characteristics of ECLIPSE participants by genotype of rs2228145

	Genotype			
	AA n (col %)	AC n (col %)	CC n (col %)	Total n (%)
**Sex**				
Male	488 (65)	659 (63)	220 (61)	1,367 (63)
Female	261 (35)	391 (37)	140 (39)	792 (37)
**Age category**				
40-<50	73 (10)	82 (8)	31 (9)	186 (9)
50-<60	195 (26)	270 (26)	91 (25)	556 (26)
60-<70	323 (43)	480 (46)	170 (47)	973 (45)
≥70	158 (21)	218 (21)	68 (19)	444 (21)
**Smoking status**				
Never	70 (9)	97 (9)	35 (10)	202 (9)
Current	232 (31)	352 (34)	115 (32)	699 (32)
Former	447 (60)	601 (57)	210 (58)	1,258 (58)
**Smoking amount (tertiles)**				
Lowest	266 (36)	344 (33)	126 (35)	736 (34)
Middle	235 (31)	312 (30)	110 (31)	657 (30)
Highest	248 (33)	394 (38)	124 (34)	766 (35)
**BMI category**				
Underweight	24 (3)	42 (4)	14 (4)	80 (4)
Normal	263 (35)	384 (37)	115 (32)	762 (35)
Overweight	284 (38)	379 (36)	136 (38)	799 (37)
Obese	178 (24)	245 (23)	95 (26)	518 (24)

BMI=body mass index

S5 Table: Summary of baseline characteristics of UK Biobank participants by genotype of rs2228145

	Genotype			
	AA n (col %)	AC n (col %)	CC n (col %)	Total n (%)
**Sex**				
Male	22,270 (47)	30,413 (47)	10,852 (47)	63,535 (47)
Female	24,962 (53)	33,836 (53)	12,055 (53)	70,853 (53)
**Age category**				
40-<50	10,333 (22)	14,112 (22)	4,899 (21)	29,344 (22)
50-<60	16,023 (34)	21,766 (34)	7,650 (33)	45,439 (34)
60-<75	20,876 (44)	28,371 (44)	10,358 (45)	59,605 (44)
**Smoking status**				
Other	41,251 (87)	56,136 (87)	20,106 (88)	117,493 (87)
Current	5,975 (13)	8,100 (13)	2,797 (12)	16,872 (13)
**Smoking amount (tertiles)**				
Lowest	19,695 (53)	27,091 (54)	9,597 (54)	56,383 (54)
Middle	6,793 (18)	9,261 (18)	3,259 (18)	19,313 (18)
Highest	10,388 (28)	13,797 (28)	4,898 (28)	29,083 (28)
**BMI category**				
Underweight	243 (0.5)	333 (0.5)	103 (0.5)	679 (0.5)
Normal	14,823 (31)	20,404 (32)	7,342 (32)	42,569 (32)
Overweight	20,256 (43)	27,244 (43)	9,676 (42)	57,176 (43)
Obese	11,781 (25)	16,073 (25)	5,735 (25)	33,589 (25)

BMI=body mass index

S1 Figure: Forest plot showing the association between rs4129267 and the risk of COPD

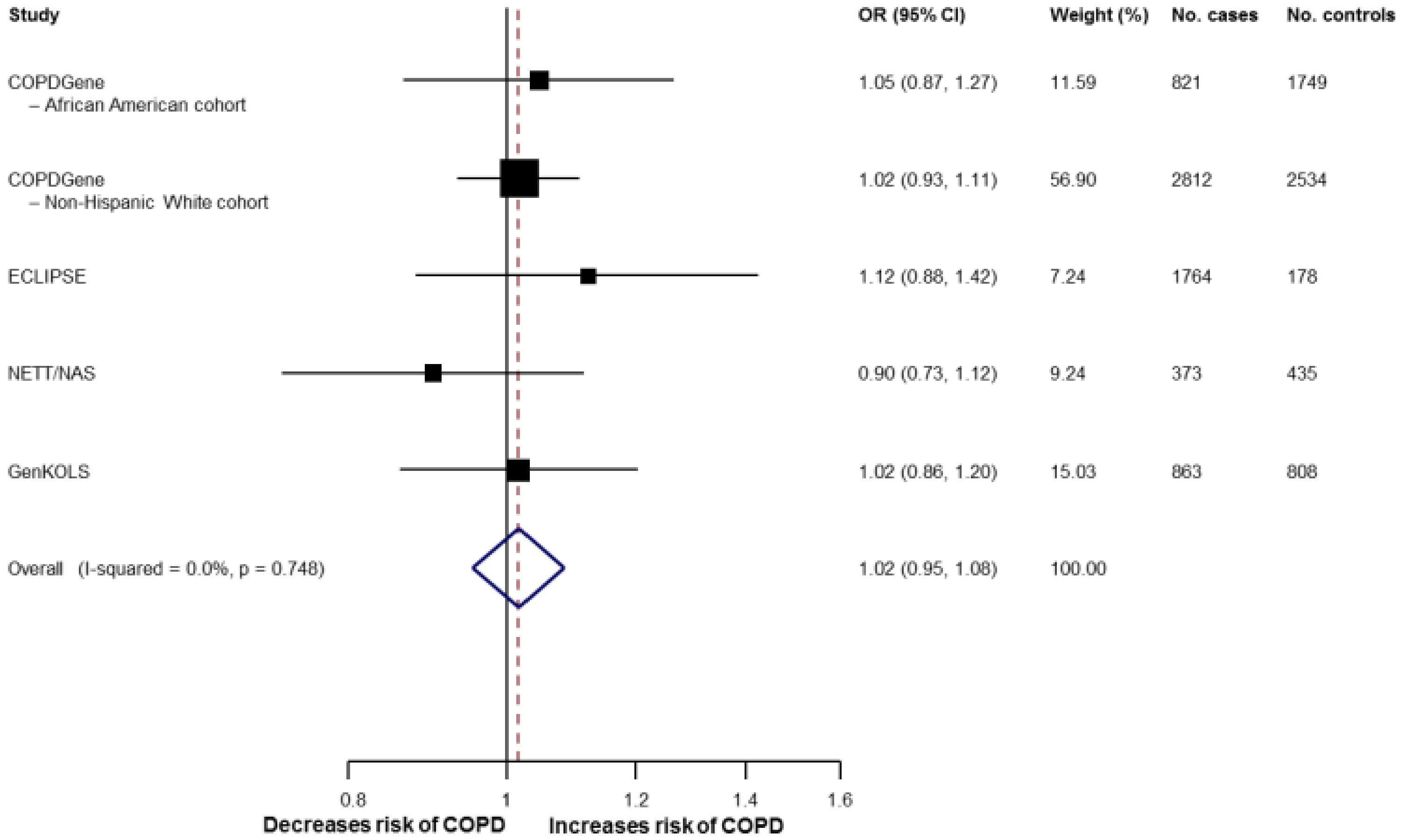

CI=confidence interval; COPD=chronic obstructive pulmonary disease; OR=odds ratio. Results are for current or former smokers and adjusted for age, ancestry principal components, and smoking amount. Fixed effects meta-analysis using inverse-variance weighting. Sizes of the boxes are proportional to the number of cases.

S2 Figure: Effect of sIL-6R trans-signaling on E-selectin, VCAM-1 expression and IL-8 release

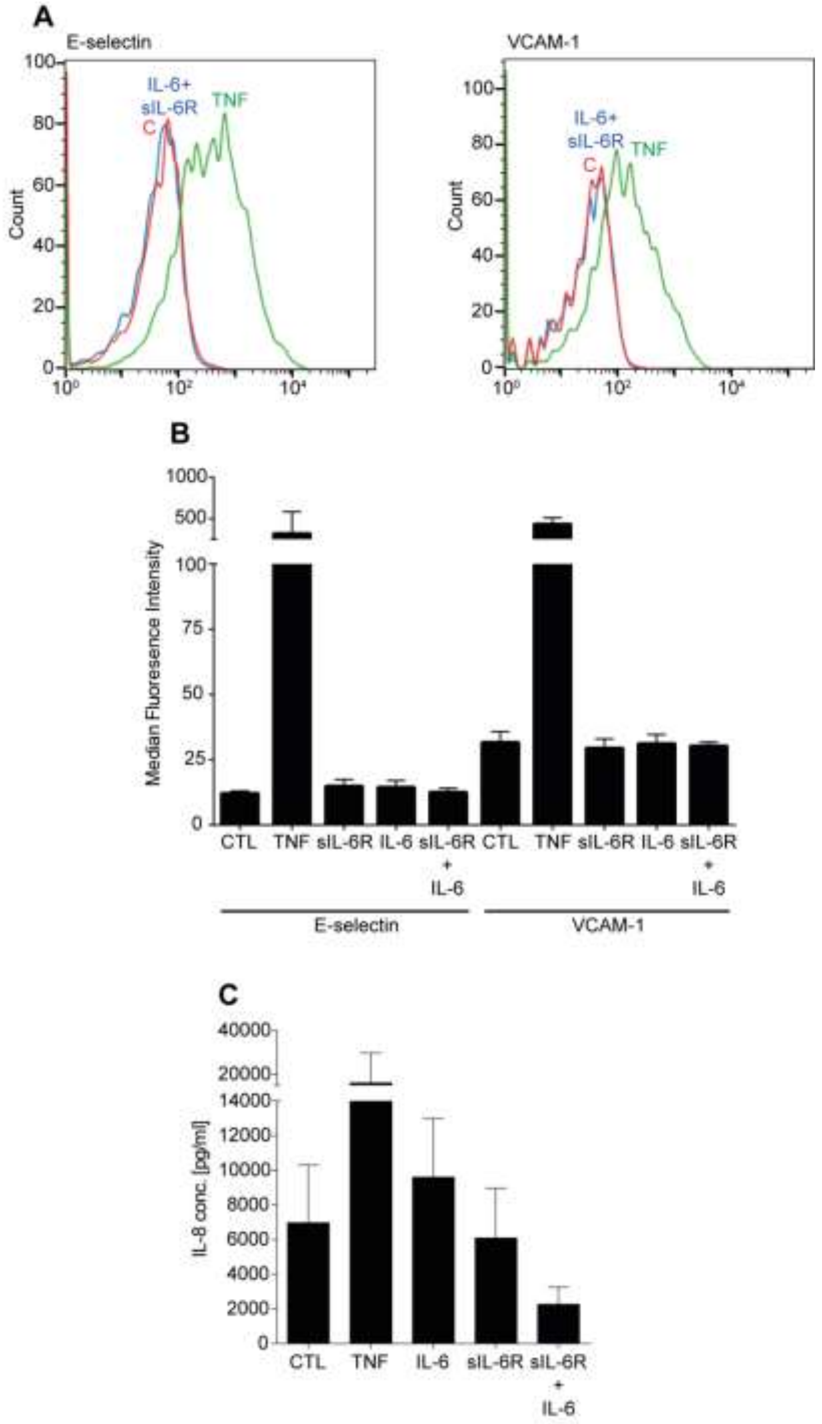

Confluent HPAECs were stimulated for 24 h with TNF-α (5 ng/ml) or sIL-6R (50 ng/ml) ± IL-6 (50 ng/ml) prior to flow cytometry as described in Materials and Methods. A. Representative flow cytometry of E-selectin and VCAM-1 expression in HPAECs after 24 h showing control (blue), TNF-α (green) and sIL-6R + IL-6 (red) treatments. B. Median fluorescence intensity of E-selectin and VCAM-1 expression. Data are representative of three independent experiments (mean ± SEM). C. Confluent HPAECs were stimulated for 24 h with TNF (5 ng/ml) or sIL-6R (50 ng/ml) ± IL-6 (50 ng/ml) prior to measurement of secreted IL-8 by ELISA. Data are representative of ≥ 2 independent experiments (mean ± SEM).

S3 Figure: Effect of sIL-6R trans-signaling on IL-8 release by HBECs

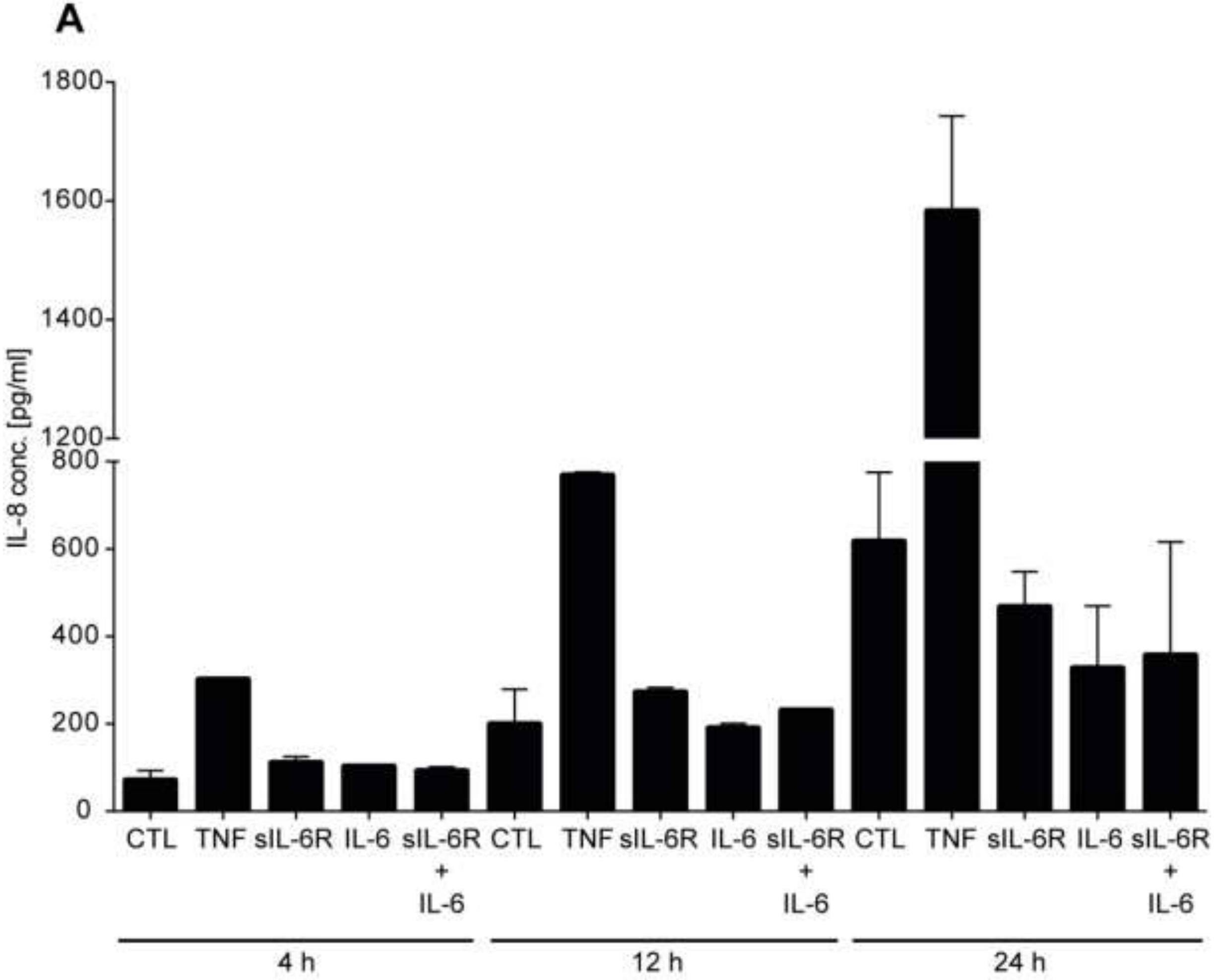

Confluent HBECs were stimulated for 4 h, 12 h and 24 h with TNF-α (20 ng/ml) or sIL-6R (50 ng/ml) ± IL-6 (50 ng/ml) prior to measurement of secreted IL-8 by ELISA. Data are representative of two independent experiments (mean ± SD).

S4 Figure: Serum from Asp358Ala carriers increases MCP-1 release from HPAECs

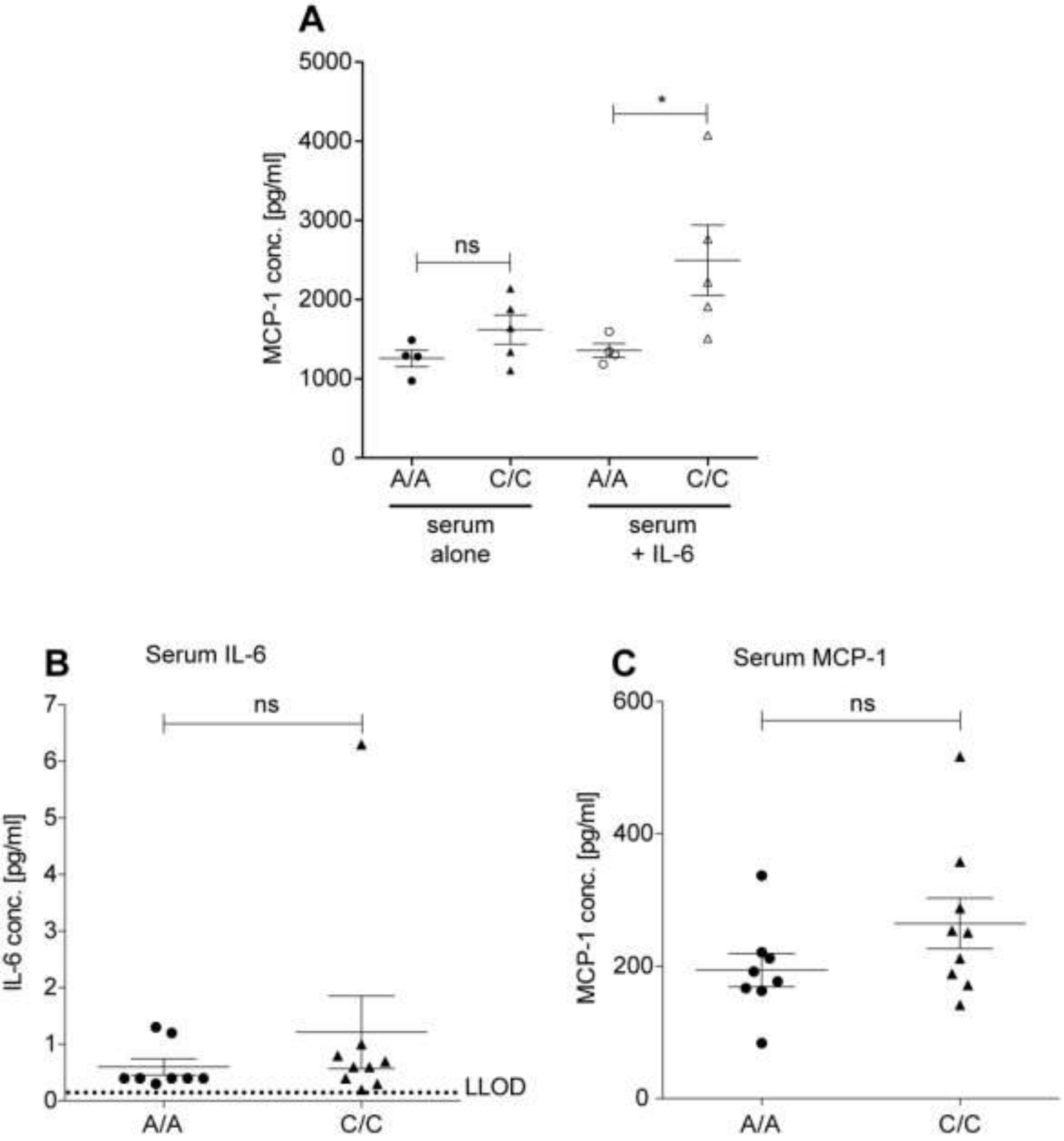

(A) Confluent HPAECs were cultured for 24 h in the presence of 65% A/A or C/C serum ± IL-6 (10 ng/ml) prior to measurement of secreted MCP-1 by ELISA. Data are presented as individual values (≥ 4) and means ± SEM. * p<0.05 using Mann-Whitney test. (B) MCP-1 and IL-6 levels in serum measured by ELISA. Data are presented as individual values and means ± SEM. LLOD = lower limit of detection.
